# Reactome diagram viewer: data structures and strategies to boost performance

**DOI:** 10.1093/bioinformatics/btx752

**Published:** 2017-11-23

**Authors:** Antonio Fabregat, Konstantinos Sidiropoulos, Guilherme Viteri, Pablo Marin-Garcia, Peipei Ping, Lincoln Stein, Peter D’Eustachio, Henning Hermjakob

**Affiliations:** 1 European Molecular Biology Laboratory, European Bioinformatics Institute (EMBL-EBI), Wellcome Genome Campus, Hinxton, UK; 2 Open Targets, Wellcome Genome Campus, Hinxton, UK; 3 Fundación Investigación INCLIVA, Universitat de València, Valencia, Spain; 4 Instituto de Medicina Genomica, Valencia, Spain; 5 NIH BD2K Center of Excellence and Department of Physiology, Medicine and Bioinformatics, University of California, Los Angeles, CA, USA; 6 Ontario Institute for Cancer Research, Toronto ON, Canada; 7 Department of Molecular Genetics, University of Toronto, Toronto ON, Canada; 8 NYU Langone Medical Center, New York NY, USA; 9 State Key Laboratory of Proteomics, Beijing Proteome Research Center, Beijing Institute of Radiation Medicine, National Center for Protein Sciences, Beijing, China

## Abstract

**Motivation:**

Reactome is a free, open-source, open-data, curated and peer-reviewed knowledgebase of biomolecular pathways. For web-based pathway visualization, Reactome uses a custom pathway diagram viewer that has been evolved over the past years. Here, we present comprehensive enhancements in usability and performance based on extensive usability testing sessions and technology developments, aiming to optimize the viewer towards the needs of the community.

**Results:**

The pathway diagram viewer version 3 achieves consistently better performance, loading and rendering of 97% of the diagrams in Reactome in less than 1 s. Combining the multi-layer html5 canvas strategy with a space partitioning data structure minimizes CPU workload, enabling the introduction of new features that further enhance user experience. Through the use of highly optimized data structures and algorithms, Reactome has boosted the performance and usability of the new pathway diagram viewer, providing a robust, scalable and easy-to-integrate solution to pathway visualization. As graph-based visualization of complex data is a frequent challenge in bioinformatics, many of the individual strategies presented here are applicable to a wide range of web-based bioinformatics resources.

**Availability and implementation:**

Reactome is available online at: https://reactome.org. The diagram viewer is part of the Reactome pathway browser (https://reactome.org/PathwayBrowser/) and also available as a stand-alone widget at: https://reactome.org/dev/diagram/. The source code is freely available at: https://github.com/reactome-pwp/diagram.

**Supplementary information:**

Supplementary data are available at *Bioinformatics* online.

## 1 Introduction

Reactome (https://reactome.org) is a free, open-source, open-data, curated and peer-reviewed knowledgebase of biomolecular pathways. It provides bioinformatics tools for visualization, interpretation and analysis of biomolecular data to support basic research, genome analysis, modelling, systems biology and education.

At the cellular level, life is a network of molecular reactions that include signal transduction, transport, DNA replication, protein synthesis and intermediary metabolism. In Reactome, these processes are systematically described in molecular detail to generate an ordered network of molecular transformations, resulting in an extended version of a classic metabolic map described by a single, consistent data model (Fabregat *et al.*, 2016). The Reactome knowledgebase thus systematically links human proteins to their molecular functions, providing a resource that functions both as an archive of biological processes and as a tool for exploring and discovering unexpected functional relationships in data such as gene expression pattern surveys or somatic mutation catalogues from tumour cells. In Reactome the steps of a pathway are represented as connected molecular events termed ‘reactions’. Reactome’s content is organized into a set of canonical pathways that corresponds to distinct biological processes with minimal overlap of reactions and proteins, arranged in a hierarchy corresponding to the GO biological process hierarchy. Each pathway is represented in a pathway diagram laid out following the Systems Biology Graphical Notation (SBGN) (Le Novère *et al.*, 2009) process description language (Fabregat *et al.*, 2016). Additionally, Reactome offers a pathway analysis service that supports enrichment and expression analysis (Fabregat *et al.*, 2016, 2017). Users can submit their own dataset for analysis and visualize the result as overlays on top of pathway diagrams.

Web browsers are one of the main types of application used for retrieving, presenting and traversing information resources on the World Wide Web. Creating an interactive pathway diagram viewer for web browsers poses a series of challenges that need to be addressed in order to offer a fast-loading and responsive product. On the one hand, implementing a custom solution enables full control over features and capabilities at the cost of longer development time. On the other hand, reusing existing software has the advantage of launching the final product in a shorter period of time but with additional features limited by the existing capabilities of the selected third party software (Krueger, 1992). Some resources like MINERVA (Gawron *et al.*, 2016) and NAVICELL (Kuperstein *et al.*, 2013) have adopted the Google map™ engine. Others such as Pathway Commons (Cerami *et al.*, 2011), WikiPathways (Kutmon *et al.*, 2016) and KEGG (Kanehisa *et al.*, 2014) developed and use their own viewers.

Reactome has always used an in-house developed diagram viewer which has evolved over the years to include enhancements in usability and performance as a response to extensive usability testing sessions aiming to improve the tool towards the needs of the community (Roto *et al.*, 2009). When the second version of the diagram viewer was released in 2013, systematic user experience testing and informal user feedback pointed out that the loading time and user interactivity needed to be improved. We describe here how we addressed these challenges, by implementing a more efficient diagram storage format, and by adopting new strategies for client data storage, retrieval and rendering. Additionally, this study aims to provide guidance to other researchers or groups working on similar visualization tools.

## 2 Implementation

The usability testing sessions showed that the users (i) had trouble using the diagram search functionality, (ii) found the diagrams too crowded/complex, especially in zoomed-out views and (iii) often lost diagram context while navigating through the event hierarchy due to the diagram’s flashing and abrupt changes of location, instead of an animated transition to the target position. Other comments highlighted the fact that the zoom was not progressive, but instead users could only zoom in predefined steps.

Aiming to address these challenges and enhance the overall user experience, a new version of the Pathway Diagram Viewer was implemented. The new version (version 3) was also focused on faster data loading, diagram rendering and element seeking. This decision was made based on the fact that users retain the feeling of being in control when an interaction between them and the computer takes no more than one second (http://www.nngroup.com/articles/powers-of-10-time-scales-in-ux).

Improvements targeted different levels and included: (i) restructuring of the data format used to send the data from the server to the client, (ii) using a graph data structure to store the pathway content on the client side, (iii) boosting the client content load strategy, (iv) implementing a multi-layer canvas approach, (v) utilising a space partitioning data structure to store the elements to be rendered and (vi) employing the delegate design pattern to control the flow of information based on the level of zoom. This section delves deeper into each aspect to describe them in finer grain.

### 2.1 Data format update

The first step to improve the overall user experience was to reduce the client loading time by replacing the eXtensible Markup Language (XML) format (https://www.w3.org/TR/REC-xml) for diagram data storage with JavaScript Object Notation (JSON) (http://www.json.org). JSON is less verbose than XML and thus has a smaller footprint. More important, JSON’s natural mapping to JavaScript objects is faster and uses fewer resources than its XML counterpart (Boci *et al.*, 2012; Nurseitov *et al.*, 2009; Wang, 2011). For all these reasons, resources that rely heavily on XML for their storage format, could potentially benefit from transitioning to JSON.

Therefore, all Reactome pathways containing diagram layout information are converted from XML to JSON and stored on the server side as static resources during the quarterly release process. In the same process, for every diagram, a graph of all the contained entities and reactions is generated and stored in an additional JSON file to enable a richer browsing and search experience throughout the diagram content. The next subsection elaborates on the creation of the graph and its usage along with the layout information.

### 2.2 Underlying graph structure

Among other elements, diagrams contain macromolecular complexes and entity sets comprised of components and members, respectively. Entity sets are used to group entities together based on common properties. Sets and complexes may have other complexes or sets as their constituents (D'eustachio, 2011). This approach quickly builds up to a highly structured network of contained entities that, in most diagrams, is conveniently represented by a single glyph that simplifies the view. Thus, the diagram viewer must be aware of all this information and able to take full advantage of it, in order to provide a much richer search function and smarter interaction with the constituents of complexes/sets.

For example, a search for a protein should highlight not only instances of the protein visible in the diagram but also any complex instances of which the protein is a part and any set of which it is a member.

**Fig. 1.**
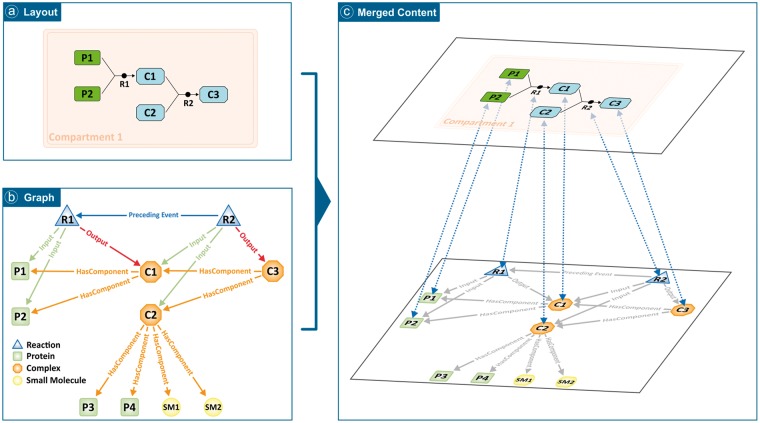
Schematic view of a pathway made up of two reactions. (**a**) The pathway diagram as presented to the final user. (**b**) Underlying graph with the whole content of the pathway. (**c**) Representation of the merging of both the diagram and graph on the client side. In the figure, P_n_ are proteins, SM_n_ are chemicals, C_n_ are complexes and R_n_ are reactions. From the graph, it can be extracted that C_1_ contains [P_1_, P_2_], C_2_ contains [P_3_, P_4_, SM_1_, SM_2_] and C_3_ contains [C_1_, C_2_], but by traversing the graph it can be easily inferred that C_3_ actually contains [P_1_, P_2_, P_3_, P_4_, SM_1_, SM_2_]

In previous versions, the client retrieved a file with the identifiers defining each element present in the diagram from the server side. In the new version, a file with a graph representing the content of the different complexes and sets for each diagram and annotating the participants of every included reaction is required (Fig. 1). This approach introduced an additional file with the graph content that has to be consumed separately by the client and merged with the layout data, once both are loaded.

The graph and layout content have elements in common, but in most cases the graph will contain more information. In the example presented in Figure 1, the pathway diagram layout contains 7 elements; 5 entities and 2 reactions (Fig. 1a), and the graph contains 11 elements; 9 entities and 2 reactions (Fig. 1b). The 4 extra elements in the graph can be justified by the fact that none of the components of C2 (4 entities) are present in the layout. Another benefit of the graph is that entities that are part of different complexes or sets are represented only once and remain accessible via graph traversing.

Because of the complementary nature of information stored in the layout and the graph files, the client side needs to implement a technique that merges both contents and allows them to seamlessly work together (Fig. 1c). Our approach is to propagate user actions from the layout level down to the graph level in order to have an easy way to traverse the content and identify the relevant entities to be highlighted by traversing up to the layout again. In addition, the built-in search feature can now take into account not only entities that are represented by a glyph in a diagram, but also all the contained entities composing that glyph. For instance, users can search among all components/members of the complexes/sets present in a single diagram. The client is able to highlight all those diagram entities containing a component (or member) that matches the search term.

For most applications that feature interactive visualizations, accompanying layout information with additional semantic metadata can prove a good practice, as it enriches the visualizations by assigning a meaning to all visual entities. In addition, this extra information can be used to enrich any existing search functionality by extending it to more than what is visualized.

### 2.3 Updated loading and caching strategies

**Fig. 2.**
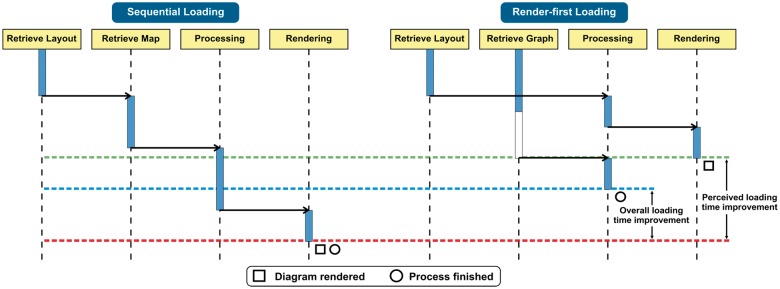
UML sequence diagram comparing sequential and render-first loading strategies. The difference between the blue and red lines shows the true loading time improvement. The improvement in the perceived loading time is highlighted by the difference between the green and red lines

The introduction of separate layout and graph files was accompanied by the adoption of a render-first loading strategy in the client (Fig. 2). The client makes concurrent XMLHttpRequest calls for the layout and the graph data content (https://xhr.spec.whatwg.org). As soon as the layout data is available, the viewer processes it and renders the diagram on the canvas. Once the graph content is ready, the latter is processed and linked to the diagram layout to be used for interactive navigation, search and future analysis overlay purposes. Following this render-first approach, the new version of the diagram viewer primes the display of the layout while it retrieves the graph behind the scenes. This strategy boosts the user experience by reducing both the true and perceived loading time.

Adopting a similar strategy that prioritizes the loading of that bit of information necessary to render something useful on the screen and, thus, engage the user, can prove particularly useful to any visualization application that requires excessive loading time. People can define a duration only when there is a clear start time and a clear end time (Seow, 2008). As a result, when users get to a point where they finally see something rendered on the screen that they can interact with, they naturally and mentally assume as the end. The rest of the loading can continue behind the scenes.

While browsing pathways, users often go back and forth among several pathways of interest, causing the viewer to load and show the same diagram several times in a relatively short period of time. A pathway diagram that has been loaded is very likely to be revisited shortly after visits to other pathways. In computer science, this is known as locality of reference (Denning, 2005), being a very clear use case for cache mechanisms. Hence, the diagram viewer implements a Least Recently Used (LRU) caching mechanism (Denning, 1968) to keep the layout and the view status (zoom level and panning) of the most recently viewed diagrams. When a diagram is revisited, the viewer does not need to request data from the server but uses the cached one in order to display the content as the user previously left it.

### 2.4 Multi-layer HTML5 canvas strategy

The new version of the diagram viewer responds to common user actions, such as hovering over an element with the mouse and selecting an entity in the diagram, by highlighting the hovered element and marking the selected entity, respectively. Aiming to provide a richer user experience and visually reinforce user actions, the diagram viewer draws a halo around the elements (reactions and participating entities) related to the selection. In addition, when the user selects an entity that is repeated in the same diagram, the viewer marks all instances of that entity as selected and draws halos around all elements related to them.

**Fig. 3.**
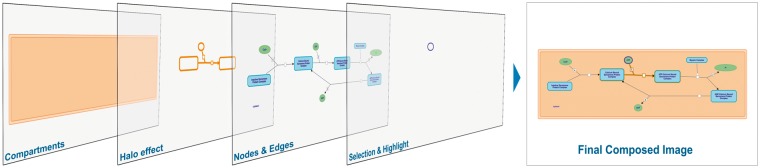
A simplified example of the adopted multi-layer canvas strategy. First four images from left to right represent different layers composing the final image: (1) Cellular compartments, (2) Halo effect, (3) Nodes & Edges and (4) Selection and Highlight. The rightmost image shows the pathway diagram as seen from the user’s perspective

To improve the visual feedback and optimize the diagram rendering process, the new version of the viewer implements a set of advanced techniques developed and used by the gaming industry. In particular, the multi-layer canvas approach (www.ibm.com/developerworks/library/wa-canvashtml5layering) was adopted to reduce the processing and redrawing overhead inherent to a single canvas update. Each of the stacked canvases in Figure 3 represents a conceptual layer and is reserved for drawing specific types of glyphs corresponding to different diagram objects such as compartments, reactions, nodes, entities or interactors. By employing this technique, only layers that require redrawing are updated, resulting in reduced rendering times in actions like highlighting or selection. This contributes to enhancing the user experience due to a more responsive behavior.

For instance, while the user moves the mouse pointer across a diagram, only the ‘Selection and Highlighting’ layer needs to be updated in order to reflect the changes in the highlighted element. Similarly, in case a diagram element is selected, only the ‘Halo effect’ and ‘Selection and Highlight’ layers need to be updated. Other resources featuring interactive visualizations that contain a lot of elements can take advantage of this strategy to improve the user experience.

### 2.5 Space partitioning data structure

Identifying the elements under the mouse pointer is a computationally demanding task if it is performed by a brute force or exhaustive search algorithm (Knuth, 1997). The cost of an exhaustive search algorithm is a linear function of the number of elements to be searched, O(n) in big O notation. Determining whether the mouse pointer position intersects with the area each element occupies can be slow, delaying the action of highlighting and making the interface appear unresponsive to the user.

To speed the search of the hovered element, our new implementation employs a space partitioning data structure, an approach often used to optimize performance. The main advantage of this data structure is that it provides a much less computationally intensive way to query for elements present in a given point or area in space, with a cost that is a logarithmic function of the number of elements to be searched O(log n) (Agarwal and Erickson, 1998).

Here, we employed a QuadTree, a tree data structure used to partition a two-dimensional space by recursively subdividing it into four quadrants or regions (Finkel and Bentley, 1974). The QuadTree is employed to efficiently (i) query only those diagram entities present in the viewport that need to be rendered and (ii) identify the entities hovered over, or selected by the mouse without having to follow the brute force method and exhaustively check every diagram object.

**Fig. 4.**
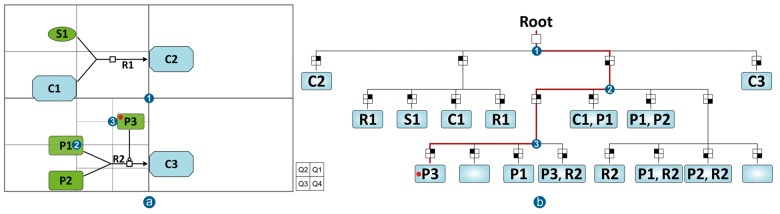
Hypothetical diagram composed of two separate reactions where (**a**) shows how the viewport is recursively split into different quadrants, so each of them contains two or less elements, (**b**) is the representation of the resulting QuadTree to achieve the two-dimensional space partitioning while (**c**) presents the same diagram elements placed in a normal collection for comparison purposes. The red dot in (a) represents the mouse pointer location and the red path in (b) depicts the tree traversing steps to narrow down the elements to be checked against the mouse location

Figure 4 provides an example of how the elements in a diagram are located in a QuadTree with quadrant size 2, meaning that only two objects are allowed per quadrant. The red line in Figure 4b highlights the path traversed in the tree to identify the element under the mouse pointer (red dot) based on a series of quick comparisons between the mouse coordinates and every quadrant center starting for the root and progressively moving down the nodes of the tree. From the root (center of the viewport) the red dot (Fig. 4a) is the 3rd quadrant (Q3); from the center of Q3 the red dot is in the first quadrant (Q1); from the center of Q1 the red dot is again in its first quadrant (Q1). Since this last quadrant is not further split, the position of the mouse pointer only needs to be compared against the contents of that quadrant, which in this case is only P3. Thus, determining that P3 is the element hovered over by the mouse pointer takes three quadrant comparisons and checking only one element of the nine present in the diagram. This provides a significant improvement over the brute force method that would check the mouse position against every element present in the diagram.

For the new diagram viewer, the QuadTree was extended to work not only with points but also with shapes that occupy diagram areas. The aim was to use it in order to narrow down the number of elements to be drawn depending on whether they are in the part of the diagram visible in the client viewport. This allows a fast, selective redraw limited to visible regions of the diagram, again improving interactivity of the diagram viewer.

Hence, the usage of this data structure could prove particularly useful for other resources featuring interactive visualizations that contain a lot of elements where the requirements include one or more of the following features: (i) determining the element hovered over by the mouse pointer, (ii) determining the selected element upon user’s click or tab action, (iii) smooth animated view transitions or (iv) progressive zoom.

### 2.6 Renderer delegates

**Fig. 5.**
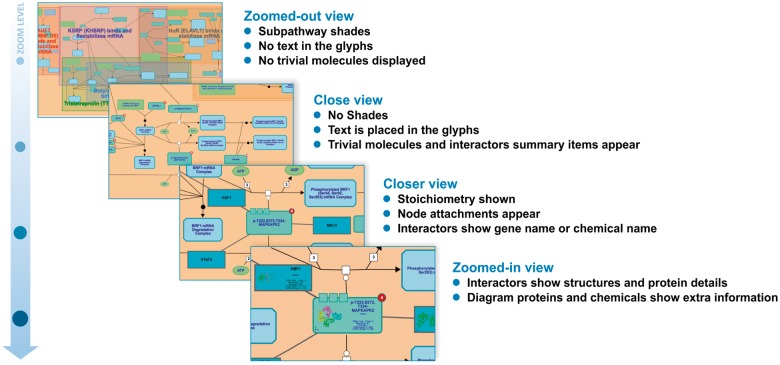
In the new pathway diagram viewer, the flow of displayed information is controlled through zooming in and out. As a result, depending on the zoom level, the viewer abstracts or enriches the view with layers of information

In order to tackle users’ requests for less cluttered pathway diagrams, but at the same time preserve access to all information stored in Reactome knowledgebase, the new viewer enables the user to control the flow of visualized information through the level of zoom. This practically means that depending on the zoom level, the viewer enriches or abstracts layers of information. Thus, each diagram entity is rendered in a slightly different way according to the level of zoom, progressively revealing more details as the user zooms in. For instance, as illustrated in Figure 5, common ‘house-keeping’ molecules, such as ADP, ATP, AMP, water, etc., are hidden in the zoomed out view, resulting in simpler and less crowded diagrams, as explicitly requested by our users.

This strategy also improves rendering time when many elements are in the viewport because fewer details are drawn. Other simplifications are to avoiding rounded corners, only showing reaction backbones without central decorators, or removing node attachments or stoichiometry. As users zoom in to specific areas, the number of elements in the viewport falls and more detail is added.

The adoption of this strategy could prove particularly useful for other resources featuring complex visualizations as it allows controlling the granularity of information displayed for a given object and level of zoom. Usability-wise, this enables resources to show different views of the same element based on the zoom, determining the optimal level of detail and type of information to be displayed in each case.

## 3 Results and discussion

The new pathway diagram viewer combines the set of strategies and data structures described above to improve performance and to include new features that aim to address the shortcomings of the previous version highlighted by the usability testing sessions. The updated diagram storage format combined with the improved ‘Render-first’ loading strategy resulted in faster loading of diagrams. Additionally, faster rendering was accomplished via the combined use of (i) a QuadTree that efficiently filters down the elements to be drawn based on the visible area, (ii) rendering delegates that declutter the view by regulating the level of detail to be drawn depending on the number of visible elements and (iii) a multi-layer html5 canvas strategy that optimizes rendering by updating only the layers that require redrawing. Optimized rendering enabled the introduction of animation and smooth transitions that, in turn, help users to maintain diagram context while navigating through pathways. The use of an underlying graph structure provided the basis for improving the built-in search feature, by including all the participating molecules of the pathway whether or not they are visible in the diagram.

Updating the underlying storage format had a positive impact on the performance of the new version of the pathway diagram viewer. To assess this performance boost, we compared the resulting file sizes for both the previous (XML) and the new data format (JSON), as well as the respective times required by the client to process them. This included the time required to populate the model in the client with the diagram data once they were retrieved from the server.

**Fig. 6.**
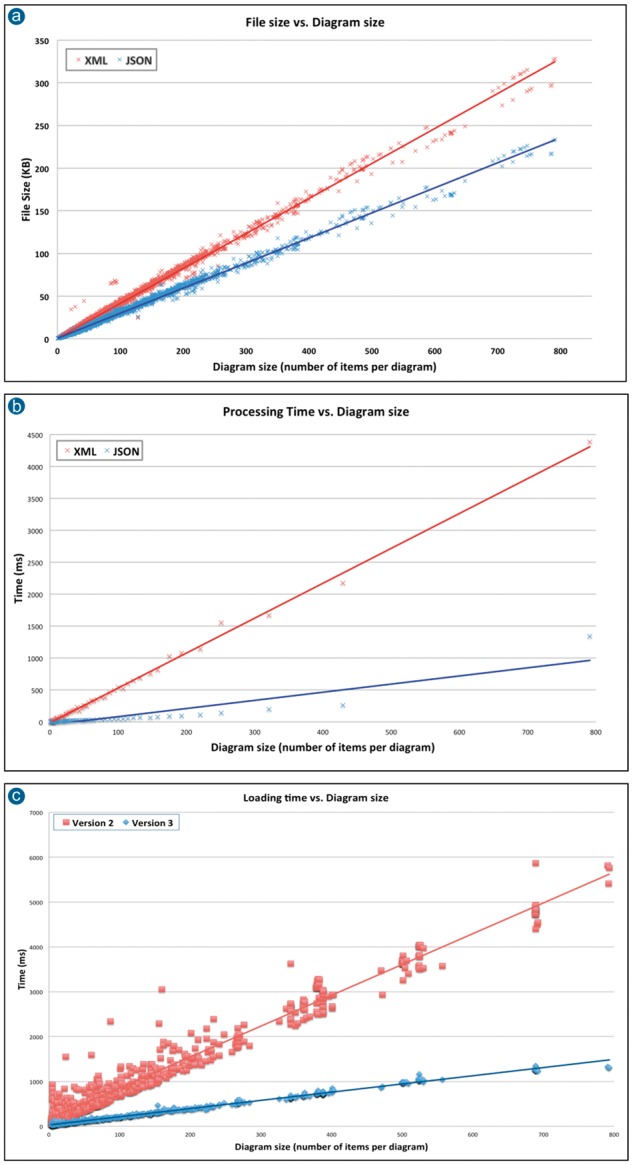
(**a**) Comparison of file sizes for both XML and JSON formats versus the diagram size in terms of number of entities present in a diagram. (**b**) Comparison of processing times achieved by Diagram Viewer v2.0 (consuming diagrams in XML) and Diagram Viewer v3.0 (consuming diagrams in JSON) versus the total number of diagram entities. (**c**) Comparison of perceived loading times achieved by Diagram Viewer version 2 (consuming diagrams in XML) and version 3 (consuming diagrams in JSON) versus the diagram size (in number of diagram entities). Measured over all human pathway diagrams from Reactome data version 52

To measure the improvement in performance, a series of experiments were conducted and the results are presented graphically in Figure 6. In particular, Figure 6a presents a chart comparing the file sizes of the Reactome diagrams against the total number of graphical entities present in them for both XML and JSON data format. As expected, for any given pathway diagram, its JSON version has a smaller file size compared to its XML version.

Figure 6b presents a comparison between the times required by the previous (2) and current (3) versions of the client to process diagrams stored in XML and JSON format, respectively, against the number of the diagram entities. The new client requires significantly less time to process any given diagram, which can be attributed to JSON’s smaller file size as well as its natural mapping to JavaScript objects, which eliminates the need for complex parsing infrastructure.

The update in the storage format combined with the new render-first loading strategy contributed to reducing the overall diagram loading time, as it is perceived by the user. This includes the time required until the diagram is loaded and fully rendered by the client. Figure 6c presents a chart comparing the times required by the previous and the new version of the client to display diagrams stored in XML and JSON format respectively against the diagram size (measured in number of entities present in a diagram).

A striking feature of the comparison of perceived loading times (Fig. 6c) is that the new diagram viewer is both faster and more consistent. One can easily notice that the times measured for the previous diagram viewer exhibit high variability, especially for smaller diagrams. This can be explained by the fact that the number of items to be drawn in diagram does not represent the actual size of the pathway in terms of participating molecules. Complexes and sets often contain several participating molecules, and encapsulated pathways might also contain a large number of participants. Also, taking into account that the top-level pathways, in the Reactome event hierarchy, are represented with diagrams which mostly contain subpathways, it is expected that they will contain a quite large number of participating molecules. This fact combined with the previous sequential loading strategy, presented in Figure 2, provides a simple explanation for those relatively small pathways, with only a few entities, that require up to 1.5 s to load. Simply put, before rendering anything on screen, the previous client had to retrieve and parse a large amount of information in order to create the map of all participating entities.

As illustrated in Figure 6c, the new version of the pathway diagram viewer achieves better performance in any given Reactome diagram. In particular, the new version of the client accomplishes loading and rendering of 97% of the diagrams in Reactome in less that 1 s (versus 57% previously); 74% of the total number of diagrams are loaded and rendered in under 0.5 s (versus 31% previously). As previously stated, keeping the application’s response times as low as possible has a positive impact on the user experience. This is particularly the case in a web application that is supposed to run inside a web browser environment, where most of its code is executed in a single thread, without use of concurrency. As a result, the adoption of the multi-layer html5 canvas strategy and the space partitioning data structure contributed to minimize CPU workload and therefore allowed room for new features such as animated transitions to be included without penalising the user’s experience.

We have conducted a usability testing session centered on the improvements described here (Supplementary Table S1). Users appreciated the animated transitions and progressive zoom functionality as they allowed for smoother and easier navigation. Users also found the new diagram viewer more responsive as it reacted to common user actions by highlighting a hovered element and marking a selected entity. Users did not express concern about crowded/complex pathway diagrams, but did react positively to the features in the new diagram that enable users to control the amount of detail displayed by zooming in and out. Regarding our improved search functionality, users made positive comments on the fact that they could now search for entities that were indirectly part of a given diagram such as members of a complex or set.

The new diagram viewer was developed in a way that can be both extended or easily integrated as it is in third party applications. Currently, Reactome offers two options for integrating this pathway diagram viewer in other web applications; either using the GWT implementation or the JavaScript wrapper. More details and examples on how to reuse this as a widget can be found at https://reactome.org/dev/diagram/.

## Conclusions

Through the use of highly optimized data structures and algorithms, Reactome has improved the pathway diagram viewer in terms of performance and usability. The new version of the diagram viewer provides a robust, scalable solution to pathway visualization that is easily integrated into third party applications.

## Funding

National Institutes of Health BD2K grant (U54 GM114833); National Human Genome Research Institute at the National Institutes of Health (U41 HG003751); European Bioinformatics Institute (EMBL-EBI); Open Targets (The target validation platform); Medicine by Design (University of Toronto). Funding for open access charge: National Institutes of Health (U54 GM114833). The funding bodies had no role in the design or conclusions of the study.

*Conflict of Interest*: none declared.

## Supplementary Material

Supplementary Data
