# Supplementary material for: Reactome diagram viewer: data structures and strategies to boost performance
Source: Bioinformatics. 2017 Nov 23;34(7):1208–14. doi: 10.1093/bioinformatics/btx752 (PMC6030826; doi:10.1093/bioinformatics/btx752)
Supplement: Supplementary Data [file btx752_supplementary_material.docx]

**Supplementary Table S1**. Summary of the addressed comments over different usability testing sessions.

| **Initial Usability Testing** | | **Action taken to address the user comments** | **Description of improvement in section** | **Latest Usability Testing** | |
| --- | --- | --- | --- | --- | --- |
| **User Comments** | **# users** |  |  | **User Comments** | **# users** |
| Diagrams are too cluttered | 2/5 | Hiding trivial molecules and showing subpathway shapes for zoomed out views, resulted in simpler and less crowded diagrams | **2.6** Renderer delegates | No negative comments about diagrams being cluttered | N/A |
| Zooming in and out is slow and clunky | 3/5 | Progressive zoom controlling the flow of information based on its level | **2.6** Renderer delegates | Appreciation of the animated transition and progressive zoom as it allowed for smoother and easier navigation | 3/6 |
| Clickable elements are difficult to find | 2/5 | Highlighting selectable elements as the mouse hovers over them | **2.2** Underlying graph structure  **2.4** Multi-layer HTML5 canvas  **2.5** Space partitioning data structure | Responsive viewer that makes it easy to identify the elements that the user can interact with | 1/6 |
| Default diagram colours difficult to distinguish | 1/5 | Created a set of colour profiles to allow user to choose between different alternatives | ** Major user interface improvement* | The availability of different colour profiles was positively commented | 2/6 |
| Search difficult to use with no comprehensive results | 3/5 | Fully reworked search feature to easily identify the type of each element in the result and show where it is in the diagram | **2.2** Underlying graph structure | Complex/set members and Reaction participants and are easy to identify. | 3/6 |
|  |  |  |  | The viewer reacts to actions in the search result panel. | 1/6 |
| Diagrams load very slow with an unclear indication of the loading status | 2/5 | New data retrieval and caching mechanisms to speed up the loading process. | **2.1** Data format update  **2.3** Loading strategy  **2.3** Caching strategy | No negative comments about the loading times | N/A |
|  |  | Messages to provide feedback of the loading status | ** Minor user interface improvement* | No negative comments about the indication of the loading status | N/A |
| Navigating the hierarchy is difficult because the diagram flashes to the reaction location | 1/5 | Smooth animated transitions were added to help users keep the context when moving across the diagram | **2.5** Space partitioning data structure  **2.4** Multi-layer HTML5 canvas | Positive comments about the animation feature | 2/6 |
